# Too little or too much: nonlinear relationship between sleep duration and daily affective well-being in depressed adults

**DOI:** 10.1186/s12888-024-05747-7

**Published:** 2024-04-25

**Authors:** Sun Ah Lee, Dahlia Mukherjee, Jonathan Rush, Soomi Lee, David M. Almeida

**Affiliations:** 1 Department of Human Development and Family Studies, The Pennsylvania State University, University Park, PA USA; 2 Center for Healthy Aging, The Pennsylvania State University, University Park, PA USA; 3 Department of Psychiatry and Behavioral Health, Penn State College of Medicine and Penn State Milton S. Hershey Medical Center, Hershey, PA USA; 4 Department of Psychology, University of Victoria, Victoria, BC Canada

**Keywords:** Sleep, Affect, Daily diary design, Depression, Major depressive disorder

## Abstract

**Background:**

In addition to having higher negative affect and lower positive affect overall, depressed individuals exhibit heightened affective reactivity to external stimuli than non-depressed individuals. Sleep may contribute to day-to-day fluctuations in depressed individuals, given that sleep disturbance is a common symptom of depression. Yet, little is known about changes in daily affect as a function of nightly sleep duration in depressed adults and non-depressed adults. The current study examined whether and how naturally-occurring sleep duration is associated with negative and positive affect, and how these associations differ between depressed vs. non-depressed adults.

**Methods:**

Data were drawn from the second wave of the National Study of Daily Experiences (NSDE), a daily diary project of the Midlife in the United States (MIDUS) study. The sample of 2,012 adults (M_age_=56.5; 57% female; 84% white) completed eight-day diary interviews via telephone on their daily experiences including nightly sleep duration and negative and positive affect. They also completed assessments of the Composite International Diagnostic Interview-Short form, and depressed status was determined based on DSM-III. Multilevel regression models with linear, quadratic, and cubic terms of sleep duration examined the nonlinear relationship between nightly sleep duration and daily affect. Interaction terms with depression status were added to examine differences between depressed and non-depressed adults.

**Results:**

Depressed adults exhibited significant and greater fluctuations in daily affect as a function of nightly sleep duration than non-depressed adults. Specifically, the degree of decrease in positive affect and increase in negative affect was greater when depressed adults slept 2 or more hours less or longer than their usual sleep hours. Non-depressed adults exhibited relatively stable daily affect regardless of their nightly sleep hours.

**Conclusions:**

Sleep duration is nonlinearly associated with affect in daily lives of depressed adults, highlighting that both having too little sleep and excessive sleep are associated with adverse daily affective well-being. Implementing sleep interventions to promote an appropriate sleep duration may help improve daily affect among depressed adults.

**Supplementary Information:**

The online version contains supplementary material available at 10.1186/s12888-024-05747-7.

## Background

Sleep disturbance is one of the symptoms of depression and an independent risk factor for recurrent depression [1]. Studies found that sleep disturbance in Major Depressive Disorder (MDD) patients was associated with poorer treatment response, increased severity of depressive symptoms, and a higher risk of suicidal ideation or attempts [2–5].Common sleep disturbance problems among depressed individuals include delayed sleep onset, early morning wakening, insomnia, and hypersomnia, which can result in both too short and too long sleep duration [6]. One study reported that individuals with depression are more likely to sleep less than 6 hours and/or longer than 10 hours [7]. Another study found that insomnia and hypersomnia symptoms co-occurred in 27% of individuals with past-year major depressive episodes, and the co-occurring symptoms were associated with more severe depression and suicide attempts [8]. Studies that examined whether sleep was predictive of subsequent depression reported somewhat mixed findings. For example, sleep indicators, including total sleep time, sleep quality, and number of awakenings, were not significantly associated with a diagnosis of depression at one-year follow-up in a female population-based sample [9], while a meta-analysis of seven prospective studies found that both shorter and longer sleep duration were associated with increased risks of depression [10]. Together, these studies suggest that both lack of sleep and excessive sleep are notable features of sleep disturbance in depressed individuals. However, how sleep duration is associated with day-to-day fluctuations of affect in depressed individuals is less explored.

Depression also disturbs mood, marked by deficient positive affect and excessive negative affect [11]. Recent studies reported that in addition to lower levels of positive mood and higher levels of negative mood in general, depressed individuals exhibited a greater variation in their mood [11] and elevated use of maladaptive emotion regulation strategies [12, 13]. Depressed individuals also showed heightened affective reactivity to daily events, such that changes in positive and negative affect in response to daily stressors and positive events were greater in depressed adults than non-depressed ones [14]. Sleep may impact different stages of emotion generation and regulation processes, such as emotional appraisals, situation selection, or situation modification [15, 16]. Inadequate sleep, either in duration or quality, was associated with less arousals in positive affect and greater arousals in negative affect in both lab-based and real-world settings [16]. The associations between sleep and affect might be more salient in depressed individuals through several potential mechanisms. Depressed individuals may have negative cognitive processes (e.g., rumination or self-criticism) more pronounced with sleep disturbances, as their cognitive resources might already be depleted while dealing with their negative emotional states [17]. Depression also involves heightened fatigue symptoms and anhedonia, loss of pleasure in generally enjoyable activities, which may impose an additional physical and emotional burden to depressed individuals in processing and regulating their emotions [18, 19]. Indeed, fatigue levels mediated the relationship between sleep quality and affect, such that lower sleep quality resulted in a subsequent increase in fatigue which resulted in a decrease in positive affect and an increase in negative affect [20]. Overlapping neurobiological underpinnings of emotion regulation and sleep (i.e., interaction of subcortical brain structures of the limbic system such as amygdala and control centers such prefrontal cortex) may also exacerbate the effects of inadequate sleep on affect among individuals with affective disorders [16, 21].

In examining the impact of sleep on affect, intensive longitudinal designs, such as daily diary designs that capture real-life experiences using repeated assessments within individuals, may be advantageous [22]. These data collection strategies could reduce discrepancies between real-time assessments and retrospective self-reports of mood, symptoms, and behaviors that change over time [23, 24]. Recent research has utilized intensive longitudinal assessments to examine differential dynamics between sleep and affect among depressed individuals and non-depressed individuals [20, 25–27]. One daily diary study noted that longer nighttime sleep duration was associated with higher positive affect the following day among youths with MDD or anxiety disorders than those without any affective disorders [26]. Another study [27] used repeatedly-measured affect, but not sleep, to examine whether habitual sleep characteristics were associated with ambulatory affect. Specifically, they focused on the person-level association between sleep quality and affect, but not day-to-day associations between two. Findings from these studies were inconclusive; studies found that the associations between sleep quality and ambulatory affect did not differ by major and minor depression diagnostic groups [20, 27], while the other revealed that sleep difficulties were differentially associated with emotional reactivity to everyday life events as a function of depression [25]. While these studies are important in taking a first step in adopting a repeated measures approach to research among a depressed population, more comprehensive analysis is warranted to examine the relationship between daily sleep and affect in depressed individuals. Further, previous studies have focused on the linear relationship between lack of sleep and daily well-being among depressed individuals. Given that both lack of sleep and excessive sleep occur in daily lives of depressed individuals [8],exploring potential non-linear associations of sleep duration with the following day’s affect is warranted.

Building upon the literature, the current study sought to examine the association between sleep duration and daily affective well-being in naturally-occurring settings, and whether this association differed by depressed status. Among various indicators of sleep (e.g., regularity, timing, quality, duration), we chose to use sleep duration considering that either too little sleep or too much sleep is the main feature of sleep disturbance in depressed individuals [7, 8]. A large body of existing literature has shown that sleep duration is a significant and robust correlate of various health outcomes, including depression, highlighting the dose-response relationship between the two [28–31]. Furthermore, sleep duration is particularly well-suited for investigating its non-linear associations with daily affect [32]. Daily diary data reporting sleep duration offers an ecologically valid means of assessing sleep quantity, effectively mitigating the recall bias often encountered in survey-based data collection methods [33]. Our focal variable of interest pertains to within-person sleep duration, which represents daily deviations from an individual’s average sleep duration over multiple days. Distinguishing itself from sleep regularity, which gauges the overall variability in sleep timing or duration across multiple days for each participant (e.g., within-person standard deviation) [34–36], this variable enables us to establish connections to daily affect. We hypothesize that there is a non-linearity in daily associations between sleep duration and affect, such that both too little and too much sleep have adverse impacts on affect and this association might be more salient among depressed individuals.

## Methods

### Participants

Data were drawn from the second wave of the Midlife in the United States (MIDUS) study and National Study of Daily Experiences (NSDE), which is a daily diary project of the MIDUS study. The MIDUS study is a national survey that aims to investigate health and well-being across middle- and older adulthood [37]. MIDUS I started in 1995–1996 which included 7,108 participants who were English-speaking US adults recruited by random digit dialing. A longitudinal follow-up was conducted in 2004–2006 (MIDUS II), which included 4,963 participants (longitudinal retention rate: 70%; mortality-adjusted response rate: 75%). Participants were contacted to participate in a baseline phone interview and then asked to complete the self-administered questionnaires by mail (SAQs; response rate: 81%). A supplemental sample mostly consisting of Black or African American participants was recruited from Milwaukee County, WI to enhance the minority representations of the MIDUS II sample. The Milwaukee sample included 592 adults who completed the baseline phone interview and SAQs (response rate: 67.2%). Then, in 2004–2009, a random subsample of MIDUS II participants was invited to enroll in the daily diary study (NSDE), resulting in a sample of 2,022 adults aged 25 to 74. NSDE participants completed telephone interviews across eight consecutive evenings and provided information about their daily experiences. A total of 14,912 interview days were completed out of a possible 16,176 days (retention rate: 92%). About 69% of the participants completed all eight days of the interviews, 88% completed at least seven interviews, and 94% completed at least six interview days. The study procedure and data documentation are publicly available at 10.3886/ICPSR04652.v8, 10.3886/ICPSR26841.v2, and 10.3886/ICPSR22840.v5.

For the current analyses, 14,811 interview days from 2,012 adults were used, after excluding 101 interview days with missing data on study variables. Participants’ mean age was 56.51 (*SD* = 12.15) ranging from 33 to 84, and 57.20% (*n* = 1,151) of them were female. The majority of the sample identified their race as White (84.4%, *n* = 1,699), 11.33% (*n* = 228) identified as Black or African American, and 4.23% (*n* = 85) identified as others. About a half (45.5%) of them completed 2 to 4 years of college or an associated degree or higher. Depressed individuals were more likely to be younger (M_age_ = 51.83 vs. 56.76 years; *t* = 6.20; *df* = 281.87; *p* < 0.001), female (72.4% vs. 55.4%; *χ²* = 22.67; *df* = 1; *p* < 0.001), and have a lower education level (college or higher = 38.8% vs. 46.3%; *χ²* = 4.39; *df* = 1; *p* = 0.036). There was no significant difference in racial groups between depressed (non-White = 15.4%) and non-depressed groups (15.6%; *χ²* = 0.00; *df* = 1; *p* = 0.954).

### Measures

#### Depression

Information on the diagnostic status of depression of participants was drawn from the main survey, where the Comprehensive International Diagnostic Interview-Short Form (CIDI-SF) [38] was implemented to assess depression status. Participants reported whether they experienced depressed mood or anhedonia in the past 12 months as well as associated symptoms, including problems with sleeping, eating, concentration, energy, feelings of self-worth, and suicidal thoughts or actions. Depression status was assigned following the criteria for MDD as defined in the Diagnostic and Statistical Manual of Mental Disorders (3rd ed., rev.; DSM-III) [39]. A diagnosis of depression was determined (1 = *yes*; 0 = *no*) when participants reported at least two weeks of either depressed mood or anhedonia “most of the day” and “nearly every day”, and experienced a series of at least four associated symptoms.

#### Daily sleep duration

During the daily nightly interviews, daily sleep duration was measured by asking a question, “Since yesterday, how much time did you spend sleeping, not including time you may have spent napping?” Responses were coded as hours and minutes on each day and the total hours of sleep were calculated (e.g., 7 hours and 30 min: 7.5 hours).

#### Daily affective well-being

##### Daily positive affect

During the daily interviews, participants were asked to rate 13 items of positive affect (feeling in good spirits, cheerful, extremely happy, calm and peaceful, satisfied, full of life, close to others, like you belong, enthusiastic, attentive, proud, active, and confident) using a five-point scale (0 = *none of the time*; 4 = *all of the time*). The average score across 13 items was used for the analyses. Within-person reliability for daily positive affect was 0.88 and between-person reliability was 0.97 [40].

##### Daily negative affect

Similar to positive affect, participants rated 14 items of negative affect (feeling restless or fidgety, nervous, worthless, so sad nothing could cheer you up, everything was an effort, hopeless, lonely, afraid, jittery, irritable, ashamed, upset, angry, frustrated) on a five-point scale (0 = *none of the time*; 4 = *all of the time*). The average rating across 14 items was used as a daily negative affect score. Within-person reliability for daily negative affect was 0.86 and between-person reliability was 0.87 [40].

#### Covariates

Age, sex (male vs. female), race (White vs. non-White), and highest education level (some college or below vs. college graduate or higher) were included as sociodemographic covariates. For daily covariates, whether any stressor occurred on a given day (stressor-day vs. non-stressor-day), whether any positive event occurred on a given day (positive-event-day vs. non-positive-event-day), and weekday (vs. weekend) were included to adjust their effects on the same-day daily affect. For daily stressors, participants reported whether each of seven types of stressors had occurred in the past 24 h: argument or disagreement, avoided an argument, stressor at work/school, stressor at home, discrimination, network stressor (i.e., stressor that happened to a close friend or family member) and any other stressor [41]. For positive events, participants reported whether each of the following five positive events had occurred each day: a positive interaction with someone, a positive event at work, a positive event at home, something good happening to a close other, and any other pleasant events [42]. Dummy-coded variables for stressor-day and positive-event-day (1 = *yes*, 0 = *no*) were used for analyses. A diagnostic status of generalized anxiety disorder based on CIDI-SF [38] and DSM-III [39] was also included as a covariate. We also controlled for person-mean sleep duration to adjust for the between-person associations of average sleep duration with daily affect.

### Analytic plan

For descriptive purposes, we first conducted Welch’s t-tests to compare statistical differences in daily sleep duration and affect between depressed and non-depressed individuals. T-tests were conducted using person-level variables aggregated to represent each person’s average values of each variable. The equality of variance was determined using Levene’s test prior to performing t-tests. We then used multilevel modeling to account for the nested structure of the data set where 14,811 daily diary days were nested within 2,012 individuals [43]. Two-level models were estimated to examine the non-linear relationship between sleep duration and affect and whether this relationship differed by depression status. Models were run separately for positive affect and negative affect. To evaluate the non-linear relationship, linear, quadratic, and cubic terms of daily sleep duration were included in the model as predictors. To test the difference of this relationship by depression status, interaction terms between these terms and depression status were included in the model (i.e., Sleep x Depression Status, Sleep^2^ x Depression Status, Sleep^3^ x Depression Status). Day-level sleep duration was centered at the person-means to interpret parameter estimates as deviations from each participants’ average sleep hours. Person-level continuous variables were centered at grand-means to evaluate estimates as deviations from the sample’s average of given variables. A sensitivity analysis was conducted by running the model using the sleep duration of which observations at the extreme level of sleep hours (i.e., less than 2 hours and more than 11 hours) were grouped together to explore the potential bias that the limited number of these observations may engender (less than 2 hours: *n*_*days*_ = 99 days, longer than 11 hours: *n*_*days*_ = 53 days). In addition, person-level daily stressor and positive events variables were included as additional covariates in a sensitivity analysis to adjust for the potential impacts of daily event variables on the relationship between sleep and affect.

## Results

### Descriptive statistics and correlations for daily variables

Table 1 shows descriptive statistics and day-level correlations between daily variables. Of 2,012 participants, 10.6% (*n* = 214) were grouped as depressed individuals who met the diagnosis criteria of depression defined in DSM-III. For average sleep duration, depressed individuals tended to sleep less than non-depressed individuals (6.94 vs. 7.15 h/day; *t* = 2.20; *df* = 246.01; *p* = 0.029). For the frequency of days with lack of sleep and excessive sleep hours, depressed individuals were more likely to sleep less than 6 h (19.3% vs. 11.7% of total interview days; *t* = -4.16; *df* = 244.86; *p* < 0.001) or longer than 9 h (6.4% vs. 3.8% of total interview days; *t* = -2.40; *df* = 240.65; *p* = 0.017) more frequently. For daily affect, depressed individuals reported lower daily positive affect (2.24 vs. 2.79; *t* = 9.17; *df* = 242.89; *p* < 0.001) and higher daily negative affect (0.43 vs. 0.17; *t* = -8.75; *df* = 225.20; *p* < 0.001) compared to non-depressed individuals. We also calculated intra-individual standard deviations (*iSD*) of sleep duration and affect and performed t-tests. Depressed individuals exhibited greater variabilities or *iSD* in sleep duration (1.20 vs. 0.93; *t* = -5.64; *df* = 237.53; *p* < 0.001), positive affect (0.44 vs. 0.33; *t* = -6.04; *df* = 235.40; *p* < 0.001), and negative affect (0.29 vs. 0.16; *t* = -8.86; *df* = 224.96; *p* < 0.001). At the day-level, sleep duration was significantly and positively correlated with positive affect, and significantly and negatively correlated with negative affect; however, these correlations were weak (*rs* = 0.05–0.07), which indicates that a linear function may not adequately explain the relationship between sleep duration and daily affective well-being.

**Table 1:** Descriptive Statistics and Day-level Correlations (*N* = 2,012; *N*_*days*_ = 14,811)

Depression and daily variable	M (SD)	Range	1	2
Depressed (*n* = 214; *n*_*days*_ = 1,485)				
1. Sleep duration	6.94 (1.83)	0–15		
2. Positive affect	2.24 (0.99)	0–4	0.07^**^	
3. Negative affect	0.43 (0.55)	0–3.5	-0.12^***^	-0.57^***^
Non-depressed (*n* = 1,798; *n*_*days*_ = 13,326)				
1. Sleep duration	7.15 (1.44)	0–18		
2. Positive affect	2.79 (0.74)	0–4	0.05^***^	
3. Negative affect	0.17 (0.28)	0–3.1	-0.08^***^	-0.44^***^

*Note*. For descriptive statistics and correlations, original values of sleep duration were used. ^**^p < 0.01, ^***^p < 0.001

### Non-linear relationship between daily sleep duration and daily affective well-being

**Fig. 1:**
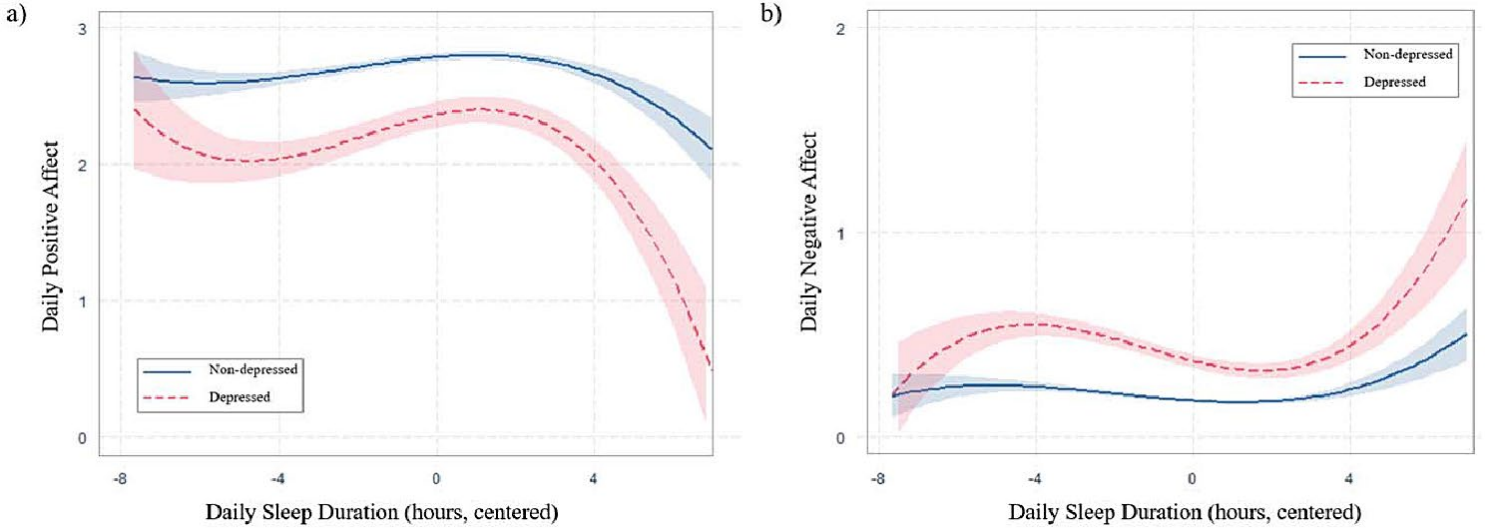
Non-linear relationship between daily sleep duration and daily affective well-being, by depressed status. Daily sleep duration was centered at person-means, such that zero indicates each participant’s average sleep duration across the daily interview days. The shaded areas represent 95% confidence intervals.

Results from multilevel models examining the non-linear relationship between daily sleep duration and daily affective well-being are presented in Table 2. For positive affect, there was a non-linear relationship between sleep duration and affect, and this association significantly differed by depression status. For non-depressed individuals, there was a curvilinear association between daily sleep duration and affect, where linear (Est. = 0.024; *SE* = 0.004; *p* < 0.001), quadratic (Est. = -0.009; *SE* = 0.001; *p* < 0.001), and cubic terms (Est. = -0.001; *SE* = 0.000; *p* < 0.001) were all significant. Depression status significantly interacted with the estimates of linear (Est. = 0.035; *SE* = 0.011; *p* = 0.002), quadratic (Est. = -0.012; *SE* = 0.003; *p* < 0.001), and cubic terms (Est. = -0.003; *SE* = 0.001; *p* < 0.001) and their effects were greater among depressed individuals than non-depressed ones (linear: 0.059 vs. 0.024, quadratic: -0.021 vs. -0.009, cubic: -0.004 vs. -0.001).This indicates that changes in daily positive affect as a function of previous night sleep duration were greater among depressed individuals. To investigate the relative importance of sleep duration and other covariates, a composite variable was created by using the estimated unstandardized coefficients for the linear, quadratic, and cubic terms of sleep duration as weights [44]. The estimated standardized coefficients indicate that the combined magnitude of the effects of sleep duration for depressed individuals equated to approximately 0.73 times the magnitude of the effect of age, which is a well-established correlate of positive affect (see Supplementary Table 1). The result was plotted in Fig. 1 to illustrate the differential effect of daily sleep duration for depressed and non-depressed individuals. Since daily sleep duration was centered at person-mean, zero on the x-axis represents each participant’s average sleep duration across all possible interview days. Figure 1(a) shows that average levels of daily positive affect were higher among non-depressed individuals (solid line) at every hour of daily sleep duration, compared to depressed individuals (dotted line). In addition, the degree of fluctuation of daily positive affect as a function of sleep hours was greater among depressed individuals. Specifically, the estimated average level of positive affect tended to increase within a range of usual sleep hours ± 2 hours (mean sleep hours: 6.94), such that longer sleep hours were associated with higher positive affect. The degree of changes in positive affect exacerbated outside of this range, where positive affect decreased when they slept 2 or more hours less or longer than their usual sleep hours. A slightly increasing trend of positive affect was also observed when they slept 5 hours less than their usual sleep hours. However, large confidence intervals around this period were found due to the small number of observations, and therefore interpretation of this result needs to be cautious. On the other hand, non-depressed individuals exhibited relatively stable positive affect, showing a slight increase and decrease outside of the range of 4 hours less or more than their usual sleep hours (mean sleep hours: 7.15).

For negative affect, there was also a non-linear association between sleep duration and affect and a significant difference by depressed status. Consistent with positive affect, the linear (Est. = -0.012; *SE* = 0.002; *p* < 0.001), quadratic (Est. = 0.004; *SE* = 0.001; *p* < 0.001), and cubic effects of sleep duration (Est. = 0.001; *SE* = 0.000; *p* < 0.001) were significant for non-depressed individuals. Depression status also significantly interacted with the estimates of linear (Est. = -0.036; *SE* = 0.006; *p* < 0.001), quadratic (Est. = 0.004; *SE* = 0.002; *p* = 0.022), and cubic terms (Est. = 0.002; *SE* = 0.000; *p* < 0.001), indicating these effects were significantly greater in depressed individuals than non-depressed ones (linear: -0.048 vs. -0.012, quadratic: 0.008 vs. 0.004, cubic: 0.003 vs. 0.001). This result suggests that changes in daily negative affect as a function of sleep duration were greater among depressed individuals. Similar to positive affect, these effects on daily negative affect among depressed individuals amounted to nearly 1.42 times the magnitude of the effect attributed to age (see supplementary Table 1). Figure 1(b) illustrates that average levels of daily negative affect were higher at every hour of sleep, and there was a greater fluctuation as a function of sleep duration among depressed individuals. Consistent with positive affect, negative affect decreased within a range of usual sleep ± 2 hours, with longer sleep hours associated with lower negative affect. The degree of changes in negative affect outside of this range exacerbated, such that when depressed individuals slept 2 or more hours less or longer than their usual sleep hours, their negative affect increased. When they slept 5 hours less than their usual sleep, negative affect decreased; however, this result may not be conclusive since there were limited number of observations around this period. Among non-depressed individuals, the estimated negative affect remained relatively stable across sleep duration, with a slight increase at 4 hours longer than their usual sleep.

**Table 2:** Results from the Multilevel Models Examining the Association between Daily Sleep Duration and Affect *(N* = 2,012; *N*_*days*_ = 14,811)

	Daily positive affect				Daily negative affect			
	Est	SE	95% CI	p-value	Est	SE	95% CI	p-value
*Fixed effects*								
Intercept	**2.825**	**0.042**	**[2.743, 2.906]**	**< 0.001**	**0.163**	**0.014**	**[0.135, 0.191]**	**< 0.001**
Daily sleep duration (linear)	**0.024**	**0.004**	**[0.015, 0.032]**	**< 0.001**	**-0.012**	**0.002**	**[-0.017, -0.008]**	**< 0.001**
Daily sleep duration (quadratic)	**-0.009**	**0.001**	**[-0.011, -0.006]**	**< 0.001**	**0.004**	**0.001**	**[0.002, 0.005]**	**< 0.001**
Daily sleep duration (cubic)	**-0.001**	**0.000**	**[-0.002, -0.001]**	**< 0.001**	**0.001**	**0.000**	**[0.000, 0.001]**	**< 0.001**
Depressed status	**-0.421**	**0.051**	**[-0.522, -0.320]**	**< 0.001**	**0.194**	**0.018**	**[0.160, 0.229]**	**< 0.001**
Daily sleep duration (linear) × Depressed status	**0.035**	**0.011**	**[0.013, 0.058]**	**0.002**	**-0.036**	**0.006**	**[-0.048, -0.023]**	**< 0.001**
Daily sleep duration (quadratic) × Depressed status	**-0.012**	**0.003**	**[-0.018, -0.006]**	**< 0.001**	**0.004**	**0.002**	**[0.001, 0.007]**	**0.022**
Daily sleep duration (cubic) × Depressed status	**-0.003**	**0.001**	**[-0.004, -0.001]**	**< 0.001**	**0.002**	**0.000**	**[0.001, 0.002]**	**< 0.001**
Average daily sleep duration	0.020	0.015	[-0.009, 0.048]	0.186	**-0.018**	**0.005**	**[-0.028, -0.008]**	**< 0.001**
Age	**0.008**	**0.001**	**[0.006, 0.011]**	**< 0.001**	**-0.002**	**0.000**	**[-0.002, -0.001]**	**< 0.001**
Gender	-0.036	0.030	[-0.095, 0.070]	0.237	-0.009	0.010	[-0.029, 0.011]	0.392
Race	-0.012	0.042	[-0.094, 0.070]	0.779	**-0.051**	**0.014**	**[-0.079, -0.024]**	**< 0.001**
Education	-0.051	0.030	[-0.111, 0.008]	0.091	-0.013	0.010	[-0.034, 0.007]	0.192
Anxiety disorder	**-0.435**	**0.107**	**[-0.645, -0.225]**	**< 0.001**	**0.271**	**0.036**	**[0.200, 0.342]**	**< 0.001**
Daily stressor (WP)	**-0.149**	**0.008**	**[-0.164, -0.134]**	**< 0.001**	**0.168**	**0.004**	**[0.160, 0.177]**	**< 0.001**
Daily positive events (WP)	**0.090**	**0.008**	**[0.073, 0.106]**	**< 0.001**	0.004	0.005	[-0.005, 0.013]	0.397
Weekend	**0.036**	**0.007**	**[0.022, 0.051]**	**< 0.001**	**-0.030**	**0.004**	**[-0.038, -0.022]**	**< 0.001**
*Random effects*								
Intercept SD	0.646				0.209			
Residual SD	0.383				0.212			

*Note*. Est = estimate; SE = standard error; CI = confidence intervals; SD = standard deviation; WP = within-person.

### Sensitivity analysis

In the sensitivity analysis, the model with sleep duration of which observations at the highest or lowest levels of sleep hours were grouped together (i.e., “2 or less hours” and “11 or more hours”) revealed that the results remained largely consistent. For positive affect, linear, quadratic, and cubic effects of sleep duration were all significant among depressed and non-depressed individuals. For negative affect, the interaction term of quadratic sleep duration and depressed status became non-significant (*p* = 0.117), indicating the quadratic effect of sleep hours in depressed individuals did not significantly differ from that in non-depressed individuals. The cubic effects of sleep duration with depressed status on negative affect remained significant.

In addition, the model with between-person daily stressors and positive events variables (i.e., the proportion of stressor-days and positive-event-days) as additional covariates revealed that the results remained the same, such that daily sleep duration was non-linearly associated with daily positive and negative affect.

## Discussion

Building upon the previous evidence that lack of sleep and excessive sleep are common symptoms of depression and related to adverse outcomes [7, 8], the current study examined how both too little and too much sleep are related to affective well-being in the daily livesof depressed and non-depressed individuals. Findings from this study showed that daily sleep duration was non-linearly related to daily affect for both groups. Having a lack of sleep and excessive sleep were associated with poorer daily affective well-being, indicated by lower positive affect and higher negative affect. Importantly, these associations were more salient among depressed individuals, suggesting that both shorter and longer sleep duration is an important indicator of sleep disturbance in the daily lives of depressed individuals.

Prior studies demonstrated a general consensus that less than 6 hours of sleep duration is inadequate for maintaining optimal health status in adults, but less consensus on excessive sleeping hours (i.e., more than 9 hours) that may be detrimental to health [45]. Findings from the current study generally support both time windows that may be important in optimizing one’s health by showing that keeping regular sleeping hours could benefit individuals’ daily affective well-being. The current study found that positive affect was the highest around their usual sleep hours, with 6.94 hours of average sleep duration for depressed and 7.15 hours for non-depressed individuals. The degree of changes in daily affect became greater when sleep duration deviated by more than 2 hours from individuals’ usual sleep hours among both depressed and non-depressed individuals. This corresponds to approximately 5–9 hours (i.e., 7 ± 2 hours) of sleep that might be optimal for maintaining pleasurable affective states in daily life. This may indicate that time windows of 6–9 hours from the prior literature could also be applied to optimizing daily well-being. Importantly, it is notable that depressed individuals might be more vulnerable to keeping these sleeping hours as they exhibited greater variability in affective responses to sleeping time.

With the 6–9 hours of time windows of sleep suggested for optimal sleep hours for health and well-being [45], depressed individuals in the current study sample reported more frequent sleep loss (i.e., sleeping less than 6 hours; 19.3% vs. 11.7% of daily interview days) and excessive sleep (i.e., sleeping more than 9 hours; 6.4% vs. 3.8% of daily interview days). This is consistent with the previous studies that found co-occurring sleep loss and oversleeping in depressed adults [8]. Since one of the diagnostic criteria for major depressive episodes is sleep disturbance (insomnia and/or hypersomnia), this result supports that both types of disturbance in sleeping hours are important indicators of depression. The prevalence of sleep loss and excessive sleep in the current study sample was somewhat lower than the previous studies [8, 46]. It might be because studies differ in measurement of sleep problems. For example, sleep complaints in one study [46] were assessed with questions asking how often they had specific sleep problems (e.g., have trouble falling asleep, wake up too early, sleep more than usual) for the last two weeks, whereas our study asked the total sleep time across eight consecutive days. Repeated measurements within individuals may assess more accurate sleep time by reducing the potential self-report bias, but our measures were not suitable for capturing the subjective quality of sleep. Future examinations could incorporate repeated assessments in measuring sleep quality in addition to sleep duration.

Daily diary studies have shown that experiencing a lack of sleep was related to impaired daily affective well-being, where shorter sleep duration was associated with lower positive affect and higher negative affect on the following day [9, 47]. Our findings are in line with these studies in that shorter daily sleep duration, especially sleeping less than 6 hours, was associated with decreased daily positive affect and increased daily negative affect. We also found a slight increase in positive affect and decrease in negative affect when participants slept much less (i.e., 5 hours) than their usual hours especially among depressed individuals. This may relate to a decreased need for sleep and an elevated mood often found in those with particular types of depression, such as manic depression [48–50]. However, this result should be interpreted with caution since the sensitivity analysis revealed that collapsing sleep hours at extreme levels (i.e., ≤ 2 hours or ≥ 11 hours) into same sleep duration did not change the results, suggesting that these observations might not account for much variation in daily affect. Nonetheless, it should be noted that depressed individuals were more responsive to having a lack of sleep than non-depressed individuals with regard to daily affective well-being, which suggests that depressed individuals might have more heightened affective reactivity to a lack of sleep. This could be because having insufficient sleep might exacerbate pre-existing mood disturbances and fatigue of depressed individuals [51]. In addition, a lack of sleep may affect neurological functions related to cognitive abilities and emotions. For example, the amygdala, of which the primary function is emotion regulation, could be impacted by having a lack of sleep, such that deprived sleep could cause a functional deficit between the amygdala and the ventral anterior cingulate cortex (vACC) which leads to decreased mood and heightened responses of the amygdala to negative stimuli [52].

The current study also found that, not only shortened sleep, but oversleeping was associated with adverse daily affective well-being, especially among depressed individuals. The results showed that sleeping 2 hours more than usual was related to decreasing positive affect and increasing negative affect, and the degrees of changes were exacerbated with an increase in sleeping hours. While the detrimental effects of oversleeping in depression were reported in a previous study including less responsivity to treatment [8], our findings suggest that daily disruption caused by oversleeping may be underlying mechanisms of these associations. The potential mechanism that oversleeping negatively impacts daily affect could be through is the disruption of circadian rhythm. Depressed individuals exhibit different patterns of variation of regional brain glucose metabolism across times of day which are correlates of diurnal mood variation [53]. Oversleeping may affect mood regulation in depressed adults with pre-existing disturbed diurnal mood variation by delaying the onset of the day or impacting wakefulness during the day. Together, given that depression is a mood disorder characterized by deficient positive affect and excessive negative affect [54], an adherence to a regular range of sleep duration in daily life could imply an important treatment implication in terms of managing daily affective well-being. In particular, we found that the effects of shorter and longer sleep duration on daily negative affect in depressed adults were nearly 1.42 times greater in the magnitude of the effect associated with age and 0.73 times greater for daily positive affect. While we cannot evade aging, we do have the capacity to modify daily sleep behaviors, thereby minimizing the adverse effects of suboptimal sleep duration. Findings from the current study underscore sleep duration as a modifiable target on a day-to-day basis for future behavioral interventions among the depressed population.

There are some limitations that should be noted. First, our results are not generalizable, especially to a clinical sample. In addition, other mood disorders (e.g., bipolar disorder) were not assessed in this community sample and thus could not be considered in the analysis. Future investigations are necessary to replicate the current study in a clinical population and with broader information on diagnostic status of other mood disorders. We also only focused on the diagnostic status of depression. Future work could extend the current study to explore potential heterogeneity based on severity of symptoms or different diagnostic groups. Such efforts could not only increase the generalizability to the clinical sample but inform future work on developing personalized treatments. Second, self-reported measures of daily sleep and affect may have potential limitations such as common-method bias [55]. Future studies using objective assessments (e.g., actigraphy for sleep measures) could reduce such bias. In addition, due to the limited data availability, sleep disorders and medication use (e.g., sleep medications or anti-depressants) were not adjusted in the current study. Controlling for the potential impacts of comorbid sleep disorders or medication use could benefit consolidating the daily associations between sleep and affect among a depressed population.

Despite these limitations, the current study builds on the literature on depression, sleep, and daily affect well-being. Our results suggest that a lack of sleep and excessive sleep were related to decreased daily affect well-being, especially in depressed individuals. These findings would provide meaningful implications for developing interventions for depressed individuals aimed at managing and encouraging healthy sleep hygiene to improve affective well-being in daily life.

## Conclusion

This study found that nightly sleep duration was significantly and non-linearly associated with daily positive and negative affect, and these associations was more salient in depressed adults. Depressed adults exhibited a greater fluctuation in daily affect as a function of sleep hours, such that when they slept 2 or more hours less or longer than their usual sleep hours, they experienced decreasing positive affect and increasing negative affect. Non-depressed adults remained emotionally stable regardless of their previous-night sleep hours. These findings highlight the importance of sleep duration as a potential target for interventions improving affective well-being among depressed individuals on a daily basis.

### Electronic supplementary material

Below is the link to the electronic supplementary material.

Supplementary Material 1

## Data Availability

The MIDUS study procedure and data documentation are publicly available at 10.3886/ICPSR04652.v8, 10.3886/ICPSR26841.v2, and 10.3886/ICPSR22840.v5.

## Abbreviations

*MDD*: Major Depressive Disorder
CIDI-SF: Comprehensive International Diagnostic Interview-Short Form
*DSM*: Diagnostic and Statistical Manual of Mental Disorders
