# Supplementary material for: Too little or too much: nonlinear relationship between sleep duration and daily affective well-being in depressed adults
Source: BMC Psychiatry. 2024 Apr 25;24:323. doi: 10.1186/s12888-024-05747-7 (PMC11044558; doi:10.1186/s12888-024-05747-7)
Supplement: Supplementary file 1 — Supplementary Material 1 [file 12888_2024_5747_MOESM1_ESM.docx]

|  | Positive affect | | Negative affect | |
| --- | --- | --- | --- | --- |
| Parameter | Standardized  coefficient | 95% CI | Standardized  coefficient | 95% CI |
| Intercept | 0.00^*^ | [0.00, 0.00] | 0.00^*^ | [0.00, 0.00] |
| Composite sleep duration | 0.09^*^ | [0.08, 0.11] | -0.08^*^ | [-0.10, -0.06] |
| Composite sleep duration × Depressed status | 0.02^*^ | [0.00, 0.04] | -0.02^*^ | [-0.03, 0.00] |
| Depressed status | -0.20^*^ | [-0.24, -0.15] | 0.25^*^ | [0.21, 0.29] |
| Average daily sleep duration | 0.03 | [-0.01, 0.07] | -0.07^*^ | [-0.12, -0.03] |
| Age | 0.15^*^ | [0.11, 0.19] | -0.07^*^ | [-0.12, -0.03] |
| Gender | -0.03 | [-0.07, 0.02] | -0.02 | [-0.06, 0.02] |
| Race | -0.00 | [-0.05, 0.04] | -0.08^*^ | [-0.12, -0.03] |
| Education | -0.04 | [-0.08, 0.01] | -0.03 | [-0.07, 0.01] |
| Anxiety disorder | -0.09^*^ | [-0.14, -0.05] | 0.16^*^ | [0.12, 0.21] |
| Daily stressor (WP) | -0.19^*^ | [-0.20, -0.17] | 0.36^*^ | [0.35, 0.38] |
| Daily positive events (WP) | 0.11^*^ | [0.09, 0.12] | 0.00 | [-0.01, 0.03] |
| Weekend | 0.04^*^ | [0.02, 0.06] | -0.06^*^ | [-0.08, -0.05] |

Supplementary Table 1.

*Standardized coefficients of fixed effects parameters from the multilevel models*

Note. Composite score of sleep duration was created based on the unstandardized coefficients as weights following Eisinga et al. (1991). ^*^ p < 0.05
